# Understanding Focal Seizures in Adults: A Comprehensive Review

**DOI:** 10.7759/cureus.48173

**Published:** 2023-11-02

**Authors:** Yash Ghulaxe, Abhishek Joshi, Jay Chavada, Shreyash Huse, Bhakti Kalbande, Prayas P Sarda

**Affiliations:** 1 Department of Community Medicine, Jawaharlal Nehru Medical College, Datta Meghe Institute of Higher Education and Research, Wardha, IND; 2 Department of Dentistry, Sharad Pawar Dental College, Datta Meghe Institute of Higher Education and Research, Wardha, IND

**Keywords:** transient ischemic attacks, epilepsy, limbic seizure, electroencephalography (eeg), focal seizures

## Abstract

Focal or partial seizures are a common neurological disorder affecting adults. This review aims to provide an in-depth understanding of focal seizures in adults, including their classification, clinical presentation, etiology, diagnosis, and management. This article seeks to enhance awareness and knowledge among medical professionals and the general public by exploring the latest research and clinical insights. Standard electroencephalography (EEG) and recordings in presurgical electrode depth in humans provide a clear definition of patterns similar to focal seizures. Models of animals with partial seizures and epilepsy mimic seizure patterns with comparable characteristics. However, the network factors supporting interictal spikes, as well as the start, development, and end of seizures remain obscure. According to recent research, inhibitory networks are heavily implicated at the beginning of seizures, and extracellular potassium alterations help start and maintain seizure continuation. An increase in network synchronization, which may be caused by both excitatory and inhibitory pathways, is correlated with the cessation of a partial seizure. Recent research on temporal lobe focal seizures in human and animal models leads to the hypothesis that the active blocking of subcortical arousal processes brings on unconsciousness. Brainstem, basal forebrain, and thalamic arousal networks' neuronal firing is diminished during focal limbic seizures, and cortical arousal can be recovered when subcortical arousal circuits are engaged. These results suggest that thalamic neurostimulation may be therapeutic to restore arousal and consciousness during and after seizures. Targeted subcortical stimulation may increase arousal and consciousness when current treatments cannot halt seizures, enhancing safety and psychosocial function for epileptic patients. We embark on an investigation into adult focal seizures in this thorough review that goes beyond a cursory knowledge of their clinical symptoms.

## Introduction and background

The hallmark of focal seizures is aberrant electrical activity in a focused location of the brain, which can cause various neurological symptoms [1]. The quality of life for those who experience these seizures can be substantially impacted by multiple underlying disorders. A sizeable portion of the adult population experiences focal seizures, sometimes called partial seizures, which pose a severe neurological issue. These seizures have a wide range of clinical manifestations because they are defined by localized aberrant electrical activity in specific brain regions. Large-scale brain networks are necessary for conscious consciousness. Despite conceptual difficulties in defining consciousness and awareness, it is evident that in healthy individuals, these extensive cortical and subcortical networks are active during ordinary conscious awareness and that neurological diseases that affect these large networks result in decreased consciousness [2,3]. The complexity of focal seizures involves not only the individuals experiencing them but also their families, caregivers, and healthcare professionals. It becomes increasingly important as our knowledge of the underlying mechanisms and management techniques grows. The distinctions between different types of focal seizures highlight how intricate these neurological events are. The additional symptoms of focal onset conscious seizures, formerly known as simple partial seizures, reflect the brain network implicated. These symptoms may include cognitive problems, autonomic alterations, sensory disturbances, and motor manifestations [3]. The clinical picture of focal onset impaired awareness seizures, formerly known as complex partial seizures, is more complex and frequently includes altered consciousness and cognitive deficits. Amnesia and other cognitive or affective alterations can occur due to static lesions of the medial temporal lobe brought on by trauma, infection/inflammation, degeneration, and other conditions without affecting consciousness [4]. Beyond their clinical manifestation, focal seizures are significant because of their genesis and underlying causes. Congenital predispositions, acquired anatomical defects, inflammatory processes, and acquired brain traumas are only a few reasons for these seizures. It is crucial to understand the intricate interactions between genetics, the environment, and neural networks to pinpoint possible therapy targets and improve management strategies.

Investigating the diagnosis and differential diagnosis of focal seizures in detail is necessary for understanding them. An interdisciplinary approach that includes a thorough medical history, physical examinations, neuroimaging methods, and electroencephalography (EEG) tests is essential for accurate diagnosis. In addition to confirming the occurrence of focal seizures, this thorough examination helps distinguish them from other medical diseases with similar clinical symptoms, such as migraines, transient ischemic attacks (TIAs), and psychogenic non-epileptic seizures (PNES). EEG data on humans revealed that during focal limbic seizures, the cortex is in a state of depression rather than stimulated. Studies using rodent models have confirmed that the cortical physiology experienced throughout focal limbic seizures is nearly similar to low-arousal states such as deep anesthesia or sleep [5]. The brain of the mouse model exhibits steady waves, hypometabolism, and decreased cerebral blood flow, identical to human limbic seizures [6-11]. Membrane potential oscillations and action potential firing patterns in cortical neurons were recorded in up and down states, simulating anesthesia and deep sleep physiology [5-8,11,12]. According to the electrochemical biosensor and genetically encoded fluorescent neurotransmitter measurements, during focal limbic seizures, thalamic and cortical transmission decreases [11,13]. The upper brainstem tegmentum displayed reduced activity on functional magnetic resonance imaging (fMRI). Focused limbic seizures occur. Direct neuronal recordings revealed decreased firing of cholinergic neurons in the nucleus basalis, pedunculopontine tegmental nucleus, probable glutamatergic neurons in the intralaminar thalamic central lateral nucleus, and serotonergic neurons in the brainstem raphe nuclei [14-16]. It is interesting to note that depending on their established cortical network connections, neurons in various thalamic regions exhibit different activity patterns during focal limbic seizures. Reduced firing of thalamic intralaminar neurons as well as subcortical cholinergic and serotonergic neurons, persistently decreased cholinergic neurotransmission, decreased fMRI signals in the central thalamus and upper brainstem tegmentum, and impaired behavioral responsiveness to cortical slow-wave activity are all characteristics of the postictal period [17]. As medical science advances, so does the range of therapeutic strategies available to manage adult focal seizures. While antiepileptic medications (AEDs) remain a cornerstone of treatment, newer modalities such as responsive neurostimulation and deep brain stimulation hold promise in modulating abnormal brain activity and improving seizure control. However, the individualized nature of focal seizures necessitates tailored treatment plans that consider factors such as the frequency and severity of seizures, underlying causes, comorbidities, and patient preferences.

In this comprehensive review, we embark on an exploration of focal seizures in adults that transcends the surface-level understanding of their clinical manifestations. By delving into the intricate classification, multifaceted etiology, diagnostic challenges, and evolving treatment landscape, we aim to equip healthcare professionals and interested readers with a holistic grasp of this complex neurological phenomenon. Through heightened awareness and deepened comprehension, we aspire to pave the way for enhanced patient care, early intervention, and improved quality of life for individuals grappling with focal seizures.

## Review

Methodology

**Figure 1:**
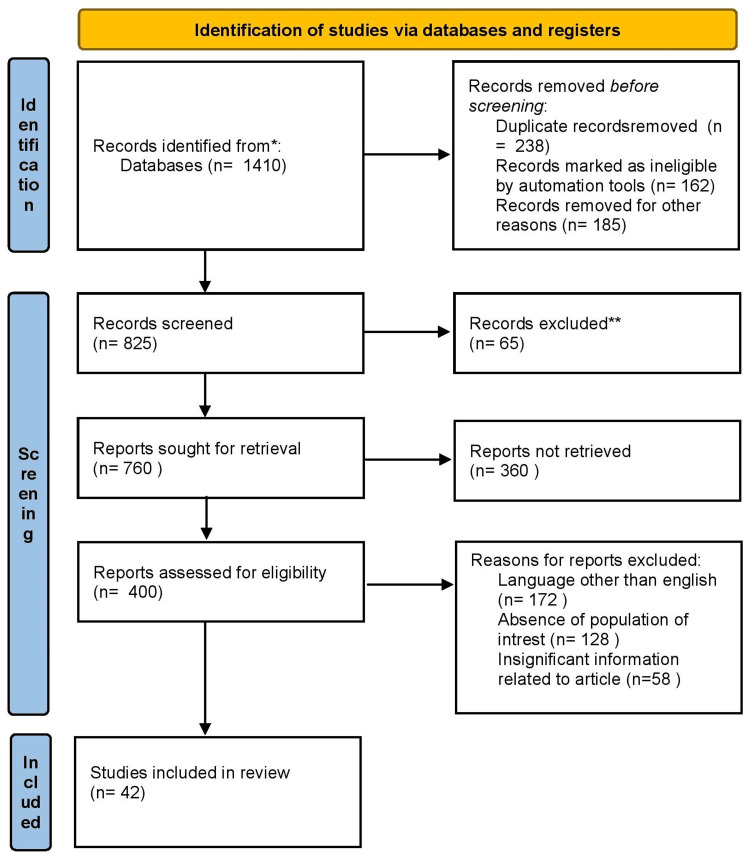
Selection process of article used in this study. Adopted from the Preferred Reporting Items for Systematic Review and Meta-Analyses (PRISMA)

We undertook a systematic search through PubMed and Google scholar in August 2023 using keywords such as “focal seizure” and “epilepsy.” The inclusion criteria were established in order to choose high-quality, pertinent studies with populations that fall within the specified age range and that cover the subjects in question in detail. Disagreements were resolved through consensus or consultation with a third-party reviewer if needed. We additionally searched for key references from bibliographies of the relevant studies (Figure 1). Reasons for exclusion of articles were as follows: different language in the article other than English, the absence of population of interest, and insignificant information related in the article.

Etiologies of epilepsy

The causes of epilepsy manifesting primarily in adulthood are mentioned in Table 1.

**Table 1:** Most typical causes of epilepsy CASPR, contact in associated protein; LGI, leucine-rich glioma inactivated; GABA, gamma aminobutyric acid; NMDAR, N methyl D aspartate

Structural	Stroke	Brain tumor	Hypoxic ischemic injury	Hippocampal sclerosis	Neurodegeneration	Malformation of cortical development
Genetic	Autosomal dominant sleep-related hyper-motor epilepsy				Familial temporal lobe epilepsy	
Metabolic	Mitochondria		Wilson disease			
Immune-mediated	Anti-CASPR2	Anti-LGI1	Anti-GABA-B	Anti-NMDA		
Infectious	Viral encephalitis		Bacterial meningitis		Fungal infections	Parasitic infections
Unknown						

There are two main categories of focal seizures: focal onset aware seizures and focal onset impaired awareness seizures. Focal seizures have a wide range of clinical symptoms. This classification scheme offers a critical framework for comprehending the varied manifestations of these seizures and how they affect people.

Focal onset aware seizures

Focal onset conscious seizures, formerly known as simple partial seizures, are characterized by limited aberrant neuronal activity within a particular area of the brain [18]. Even though these seizures have specific neurological symptoms, consciousness is unaffected. The symptoms of focal onset conscious seizures show how intricately the brain is mapped and how intricately it interacts with the body. Motor manifestations in focal onset conscious seizures may be accompanied by mild or severe motor alterations. They range from solitary jerks or twitches of particular muscular groups to more intricate motions such as automatisms or repetitions. These motor manifestations are caused by abnormal motor area activation, which briefly impairs regular motor control. Sensory disturbances are another aspect of focal onset conscious seizures. Changes in sensation, such as tingling, numbness, or perceptual abnormalities, may occur in people. These sensory phenomena show the complex somatosensory map of the brain as a result of aberrant neuronal firing within sensory processing regions. In addition to focal onset aware seizures, autonomic changes might affect physical processes that are usually controlled automatically. Variations in heart rate, blood pressure, and even gastrointestinal feelings may be a part of these shifts. These unique autonomic responses result from the extensive connections between the afflicted brain area and autonomic centers. Focal onset conscious seizures can also subtly affect cognitive processes, according to research. People occasionally have brief bouts of cognitive changes, such as déjà vu (the sensation of having already experienced something) or jamais vu (the feel of a familiar situation being foreign). The brain areas involved in memory, familiarity, and cognitive processing are the source of these cognitive disturbances.

Focal onset impaired awareness seizures

Focal onset impaired awareness seizures, formerly known as complex partial seizures, are characterized by a more complex interaction between aberrant neural activity and altered consciousness. Contrary to focal onset conscious seizures, people having these seizures frequently show less awareness of their environment and may make aimless or partially purposeful motions. The alteration of consciousness is a defining feature of focal onset seizures with reduced attention. There may be a blank stare, a change in how they react to outside stimuli, or even a total loss of awareness. These changes result from aberrant electrical activity spreading to associated brain areas, sustaining alertness and cognitive engagement. Semi-purposeful motions in focal onset seizures with reduced consciousness frequently include automatisms, which are repetitive, semi-purposeful motions. Lip-smacking, plucking at clothing, fumbling, and other actions are automatic and uncontrolled. The complex linkages between the afflicted brain areas and the motor centers are reflected in automatisms. The postictal condition, which can occur after focal onset impaired awareness seizures, is characterized by confusion. Cognitive fog, haze, and memory difficulties describe this condition. The brain temporarily struggles to re-establish homeostasis following the pungent neuronal discharge during a seizure, as shown in the postictal state [18].

Clinicians can diagnose patients and devise effective treatment plans by using the two aforementioned separate classifications of focal seizures. Healthcare providers can better customize their interventions to meet the unique requirements of people suffering from these seizures by understanding the distinctive clinical manifestations connected with focal onset aware seizures and focal onset impaired awareness seizures. Depending on the part of the brain that is damaged, focal seizures can have quite different clinical presentations. Symptoms include sensory problems, autonomic alterations, motor movements (twitching or repetitive activities), and cognitive deficits. The symptoms frequently reflect the affected brain region and can occasionally be confused with those of other neurological or mental illnesses. Infections, head injuries, infections, anatomical abnormalities of the brain (such as tumors or scars), genetic susceptibility, and cerebrovascular diseases are only a few causes of focal seizures. For effective therapy and management, it is essential to determine the underlying cause. A thorough evaluation is necessary to make an accurate diagnosis of focal seizures, which includes a comprehensive medical history, a physical examination, and neuroimaging procedures (such as MRI and CT scans) to look for any structural abnormalities such as low-grade tumors and malformations of cortical development. To record the aberrant electrical activity in the brain, EEG is essential. Partial seizures are self-terminating occurrences generally lasting 2 to 6 minutes [18]. Depending on the method of recording, human electrographic patterns can be described in varying degrees. Scalp EEG recordings help identify the onset patterns and location of seizures. Still, they are not the best method for detecting seizure activity across the entire cortical, ipsilateral, and contralateral regions of epilepsy [19]. Technically, simple scalp EEG monitoring reveals partial seizures coincide with rhythmic, high-amplitude activity. At the end of the seizure, focal postictal slowing with substantial amplitude activity in the frequency range is frequently observed. A flattening of the EEG signal and the emergence of decreased amplitude and quick rhythms are indicators that partial seizures are beginning to be recorded on the scalp [20,21]. Surgery can be used to locate the epileptogenic area, and long-term intracranial recordings are more precise than EEG in describing patterns of seizure close total discharge producer. Seizure patterns observed in various lobes and cortical regions do not significantly vary. Among the seven forms of patterns of intracranial EEG found in a population of individuals with partial epilepsies brought on by various causes, ictal and interictal patterns were most frequently seen [22]. Type IIb localized cortical dysplasias, periventricular heterotopias, and mesial TLE with substantial cell loss and gliosis have all been associated with ictal patterns that are unique to the underlying epileptogenic lesion [23-25].

"With time, the patterns of human partial seizure changes" in contrast to large amplitude EEG oscillations made by generalized idiopathic epilepsy (such as spike and wave complexes) that essentially remain unchanged throughout the seizure [26]. The most prevalent indicator of the beginning of a seizure, according to scalp EEG recordings of humans, is a decrease in background activity along with the emergence of rapid activity in range (also known as flattening of EEG) [21]. Clinical studies based on long-term intracranial depth recordings from the epileptogenic zone during presurgical stereo-EEG monitoring have shown that the common sequence at the beginning of seizures is characterized by a loss of background activity and a substitution of this with low-voltage fast activity at 20-100 Hz, which is usually superimposed to slow potential [27-29].

The vast majority of studied partial epilepsies are found to have low-voltage rapid activity, which has a critical localizing/lateralizing value [30,31]. Most intracranially investigated partial epilepsies, including focal cortical dysplasias and postanoxic lesions, exhibit this EEG pattern [32]. Large-amplitude spike potentials are a characteristic of the hypersynchronous pattern, another typical seizure-onset pattern [33]. The hypersynchronous pattern has never been seen in neocortical focal epilepsies, but it is most frequently observed in TLEs with hippocampal sclerosis; other, less frequent patterns of seizure onset are categorized as far fields, which are volumetric data collected from cortical sources [22]. Therefore, epilepsy-causing networks cannot be localized by these patterns. The mesial TLE model of animals and acute temporal seizures model show hypersynchronous seizure-onset patterns and low-voltage fast activity [34,35]. These two patterns might indicate the same networks being activated due to large-amplitude spikes that are regularly seen within hypersynchronous patterns usually accompanied by brief runs of low-voltage fast activity. Both patterns, according to experimental studies, are created by the limbic system in the in vivo pilocarpine model of mesial TLE and in vitro entire cortex of guinea pig stimulated with various convulsive medications, such as 4-aminopyridine or bicuculline [36]. Partially recorded intracranial partial seizures in both human and animal models consistently show a transition toward a tonic discharge after the onset of a seizure, which is identified by low-voltage rapid or hypersynchronous patterns. As per the alternate hypothesis of potassium chloride co-transporter 1 (KCC2), high interneuronal activity on network excitability may be a cause of this transition due to changes in extracellular potassium. The German school’s investigations demonstrated that extracellular potassium increases are correlated with the start of seizures [37]. Later studies demonstrated that inhibitory network activation is enough to induce significant variation in extracellular potassium concentration. As previously reported, the neuronal potassium chloride (KCl) cotransporter, which enabled KCl extrusion from main neurons during GABAergic inhibition, also contributes to an inhibitory network-driven rise in extracellular potassium [38]. This theory holds that inhibitory network activity and subsequent release of GABA by interneurons significantly stimulated GABA type A receptors, causing chloride to build up inside cells and for KCC2 to extrude potassium. By decreasing the electrochemical drive of chloride ions, the rise in extracellular potassium modulates inhibition and lessens inhibitory constraint seen at the onset of seizure. In the study by Trombin et al., the change from low-voltage rapid activity to tonic firing in the brain of an isolated guinea pig was connected with increases in extracellular potassium [39]. According to this study’s findings, high potassium levels could cause main neurons to fire erratically. Ectopic firing, along with a drop in inhibition, may strengthen the excitatory contacts between the main cells that mutually recruit and produce firing commonly seen at tonic discharge. All partial seizures outlined in epileptic patients or animals, as well as acute seizures induced by medication, show a variation from tonic firing into rhythmic bursting (also known as the “clonic” phase of the seizure, a vague term came from the clonic contractions considered at the end of a generalized seizure). The gradual synchronization of neuronal activity that takes place when primary neurons are stimulated throughout the tonic phase may be the cause of this tonic-to-bursting transition. At the end of a partial seizure, large amplitude activity with large amplitude bursts that are synchronized in time and spatially frequently emerges, strongly indicating that excitation sync may be implicated at the end of the seizure [40]. Human partial seizures have been shown to resynchronize intracranial signals during the late seizure phase. This research suggests that networks may be split at seizure initiation, integrate as the seizure progresses, and then converge into a single dominating component just before the seizure ends [41].

Distinguishing focal seizures from other conditions, such as migraines, TIAs, and PNES, is essential due to the similarities in symptoms. Clinicians must employ various diagnostic tools to ensure an accurate diagnosis. Treatment strategies for focal seizures include AEDs, lifestyle modifications, and, in some cases, surgical intervention. The choice of treatment depends on factors such as seizure frequency, severity, underlying cause, and individual patient characteristics. Traditional methods of treating focal seizures often involve the use of AEDs as the first line of treatment. These medications are prescribed by a healthcare professional based on the individual’s specific seizure type, medical history, and overall health. New therapeutic options for focal seizures are being investigated as a result of advancements in medical research. These include neuromodulation methods that target and control aberrant brain activity, such as responsive neurostimulation and deep brain stimulation. Currently, a treatment option is available for people with temporal lobe epilepsy (TLE) where surgical resection ’is not possible. Neurostimulation of the medial temporal lobe and stimulation of deep brain anterior thalamic nuclei targeting subcortical arousal regions with neurostimulation could be applied to ictal and postictal periods to reawaken arousal and consciousness [42]. With appropriate diagnosis and management, many individuals with focal seizures can achieve good seizure control and an improved quality of life. However, the prognosis varies depending on factors such as the underlying cause, response to treatment, and any associated comorbidities. A summary of all the articles included in this review is listed in Table 2.

**Table 2:** Summary of articles included in the review. EEG, electroencephalography; FDG-PET, fluorodeoxyglucose-positron emission tomography; GABA, gamma aminobutyric acid; ISWs, initial slow waves; SEEG, stereo-electroencephalography

Author	Year	Findings
Koch et al. [1]	2016	It studies the relationship between specific patterns of brain activity and conscious experiences.
Dehaene et al. [2]	2017	It discusses the mechanisms and criteria that might be associated with consciousness in humans.
Laureys et al. [3]	2015	Covering various aspects and disorders related to consciousness.
Squire et al. [4]	2011	The inquiry into how distinct structures within the medial temporal lobe may have varying roles in the functions of memory is a central concern in the field of neuroscience.
Englot et al. [5]	2008	It investigates how such seizures can impact the neocortex and provides insights into the spread and consequences of seizures in the brain.
Englot et al. [6]	2009	It sheds light on the interactions between different brain regions during seizures and their impact on cortical activity.
Kundishora et al. [7]	2017	It investigates deep brain stimulation to restore conscious arousal during focal limbic seizures.
Sieu et al. [8]	2021	Mouse model of focal limbic seizures reproducing behavioral impairment and slow wave activity.
Sieu et al. [9]	2022	Presented a mouse model of electrically inducible focal seizures with impaired consciousness.
Gummadavelli et al. [10]	2021	Demonstrated a correlation between cortical low-frequency power and behavioral impairment in a seizure model.
Motelow et al. [11]	2015	Decreased subcortical cholinergic arousal during focal seizures, providing insights into epilepsy.
Yue et al. [12]	2020	The activity of cortical neurons during focal limbic seizures, focusing on “up and down” states.
Sieu et al. [13]	2021	Mouse model of focal limbic seizures with impaired behavior, cortical slow waves, and reduced cholinergic arousal.
Feng et al. [14]	2017	Thalamic activity patterns during focal limbic seizures, highlighting divergence in thalamic responses.
Zhan et al. [15]	2016	Explored impaired serotonergic brainstem function during and after seizures, implicating serotonin in seizure dynamics.
Andrews et al. [16]	2019	Mechanisms of decreased cholinergic arousal in focal seizures using in vivo whole-cell recordings.
Wu et al. [17]	2014	Ictal baseline shifts and high-frequency oscillations in mesial temporal lobe seizures using SEEG.
Jenssen et al. [18]	2006	Duration of seizures recorded in the epilepsy monitoring unit.
Pacia et al. [19]	1997	Intracranial EEG substrates of scalp ictal patterns from temporal lobe foci, offering insights into seizure localization.
Gloor [20]	1975	Discussed the electroencephalography and electrocorticography in the neurosurgical treatment of epilepsy.
Fariello et al. [21]	1979	Generalized cortical electrodecremental event observed in patients with dystonic seizures.
Perucca et al. [22]	2014	Intracranial EEG seizure-onset patterns and their relationship with underlying pathology.
Tassi et al. [23]	2002	Focal cortical dysplasia, examining neuropathological subtypes, EEG, neuroimaging, and surgical outcomes.
Spencer and Pappas [24]	1992	Surgical decisions regarding medically intractable epilepsy, providing insights into epilepsy management.
Ogren et al. [25]	2009	Presented three-dimensional hippocampal atrophy maps that distinguish common temporal lobe seizure-onset patterns.
Gnatkovsky et al. [26]	2014	Biomarkers of the epileptogenic zone using quantified SEEG analysis, aiding in the localization of seizures.
De Curtis and Avoli [27]	2015	Initiation, propagation, and termination of partial (focal) seizures, contributing to seizure understanding.
Allen et al. [28]	1992	High-frequency rhythmic activity during SEEG suppression in frontal lobe epilepsy, relevant to seizure analysis.
Fisher et al. [29]	1992	EEG activity at the start of seizures, contributing to the understanding of seizure onset.
Gotman et al. [30]	1995	Frequency of electroencephalographic discharge in seizures of focal and widespread onset, focusing on intracerebral recordings.
Pelliccia et al. [31]	2013	Ictal EEG modifications in temporal lobe epilepsy, providing insights into the electrophysiological aspects of seizures.
Engel et al. [32]	1990	Presurgical evaluation for partial epilepsy, comparing the contributions of chronic depth-electrode recordings, FDG-PET, and scalp-sphenoidal ictal EEG.
Maillard et al. [33]	2004	Semiologic and electrophysiologic correlations in temporal lobe seizure subtypes, aiding in seizure classification.
Bragin et al. [34]	2007	ISWs at seizure onset in patients with drug-resistant temporal lobe epilepsy, contributing to understanding seizure dynamics.
Boido et al. [35]	2014	Simultaneous enhancement of excitation and post-burst inhibition at the end of focal seizures, providing insights into postictal phenomena.
Carriero et al. [36]	2010	Epileptiform discharge patterns in the olfactory and limbic areas of the isolated guinea pig brain during treatment with 4-aminopyridine.
Heinemann et al. [37]	1977	Extracellular free calcium and potassium levels during paroxysmal activity in the cerebral cortex of cats, contributing to our understanding of ion dynamics during seizures.
Viitanen et al. [38]	2010	K+-Cl cotransporter KCC2 in promoting GABAergic excitation in the mature rat hippocampus, shedding light on GABA-mediated excitation.
Trombin et al. [39]	2011	Action potential features during focal seizure discharges in the entorhinal cortex
Jiruska et al. [40]	2013	Synchronization and desynchronization in epilepsy, offering perspectives and hypotheses on these complex processes.
Kramer et al. [41]	2012	Human seizures self-terminate across spatial scales via a critical transition.
Geller et al. [42]	2017	Brain-responsive neurostimulation in patients with medically intractable mesial temporal lobe epilepsy.

## Conclusions

Adults who experience focal seizures have a complex neurological condition with a variety of clinical manifestations and underlying causes. For maximizing results and enhancing the general well-being of those who are affected by these seizures, early diagnosis, correct classification, and individualized treatment strategies are essential. In conclusion, this study illuminates the complex nature of focal seizures in adults and emphasizes the significance of a thorough approach to diagnosis and treatment. Healthcare practitioners may give patients with focal seizures the greatest care by remaining up to date on the most recent developments in research and therapy. We have presented experimental data that unequivocally highlight the crucial roles of both inhibitory and excitatory synaptic transmission within neural networks, which include inhibitory interneurons and primary glutamatergic neurons. However, additional pathways might be crucial to epileptiform synchronization.
